# A UWB-Based Lighter-Than-Air Indoor Robot for User-Centered Interactive Applications

**DOI:** 10.3390/s22062093

**Published:** 2022-03-08

**Authors:** Khawar Naheem, Ahmed Elsharkawy, Dongwoo Koo, Yundong Lee, Munsang Kim

**Affiliations:** Center for Healthcare Robotics, School of Integrated Technology, Gwangju Institute of Science and Technology (GIST), Gwangju 61005, Korea; kkkhawar@gm.gist.ac.kr (K.N.); elsharkawy@gm.gist.ac.kr (A.E.); dongwoo.koo@gist.ac.kr (D.K.); dldbsehd278@gist.ac.kr (Y.L.)

**Keywords:** autonomous vehicle, control, human–robot interaction, indoor tracking, UWB sensor, wearables

## Abstract

Features such as safety and longer flight times render lighter-than-air robots strong candidates for indoor navigation applications involving people. However, the existing interactive mobility solutions using such robots lack the capability to follow a long-distance user in a relatively larger indoor space. At the same time, the tracking data delivered to these robots are sensitive to uncertainties in indoor environments such as varying intensities of light and electromagnetic field disturbances. Regarding the above shortcomings, we proposed an ultra-wideband (UWB)-based lighter-than-air indoor robot for user-centered interactive applications. We developed the data processing scheme over a robot operating system (ROS) framework to accommodate the robot’s integration needs for a user-centered interactive application. In order to explore the user interaction with the robot at a long-distance, the dual interactions (i.e., user footprint following and user intention recognition) were proposed by equipping the user with a hand-held UWB sensor. Finally, experiments were conducted inside a professional arena to validate the robot’s pose tracking in which 3D positioning was compared with the 3D laser sensor, and to reveal the applicability of the user-centered autonomous following of the robot according to the dual interactions.

## 1. Introduction

In recent years, there has been a noticeable and remarkable interest in using robots for indoor applications where robots and humans share the same space. However, indoor spaces are often accompanied by certain technical challenges such as the absence of a GPS signal, the presence of ambient light conditions [1] and electromagnetic disturbances [2,3], and thus have demanded the adoption of better solutions for tracking, controlling, and navigating robots indoors [4]. Unlike ground robots, indoor flying robots are helpful in many fields, e.g., inventory logistics, visual inspection, interactive movie-making, etc., as they usually occupy unused over-head space and collaborate with humans in more flexible ways [5,6]. However, typical flying robots, such as drones, can be harmful due to their powerful propellers when required to fly in close proximity to people [7]. Furthermore, they generally cannot fly for a long time without recharging and cannot carry a relatively heavy payload [8]. According to a study [9] on human–robot interaction (HRI) with flying robots, the lighter-than-air robots (LARs) are more accepted by humans than drones as they generate less noise and have a friendly appearance.

In this work, we used a special type of LAR called lighter-than-air indoor robot (LAIDR) which comprises helium gas contained within a spherical poly-vinyl chloride (PVC) hull. The LAIDR is equipped with modular 3D printed parts, making it easier to upgrade and maintain. The LAIDR carries a relatively heavier payload owing to helium lifting, which is considered sufficient to carry a camera, gimbal, and other helpful peripherals.

**Figure 1:**
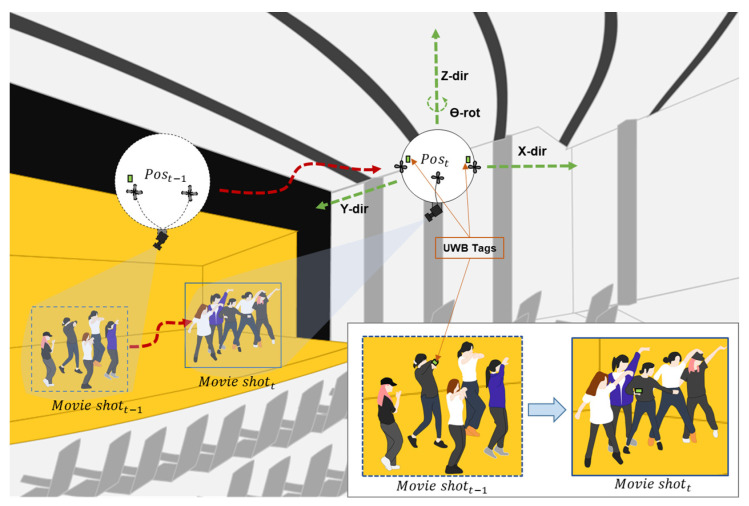
Illustration of the target application where the UWB-based LAIDR acts as an indoor movie-making agent during a live show and captures specific actor movie shots by automatically following the actor’s UWB position.

Researchers have introduced various uses for indoor autonomous LARs in social applications such as education [10,11], entertainment [12,13], human following [14,15], and teleoperation [16]. Each LAR has unique design aesthetics and tracking modalities. The authors [10,11] developed an open-source automated LAR for robotics education and research purposes. However, their designs bear a low payload capacity. The authors of [12,13] presented an automated LAR for an interactive entertainment application in a large indoor space. Although their designs were robust and carried more payload, they applied computer vision to interact with the audience which may cause the robot to react poorly under indoor variable light conditions, and equipped the user with multiple wearable sensors to detect user intention for HRI which not only increases the user’s workload owing to multiple wearables but also fails to localize the user. In [14,15], the first HRI application for indoor human following with an automated LAR was introduced where an on-board monocular camera detects faces, recognizes hand gestures, and follows a person. However, during interaction the user must always remain near the flying robot, which means this solution cannot be used for long-distance user interaction in larger indoor areas. Another study [16] employed the automated LAR for teleoperation-based indoor exploration and auto-landing. However, this study used visual feedback provided by the LAR’s on-board camera for teleoperation and visual marker-based pose estimation for auto-landing. As evident from previous work, camera- and wearable-sensor-based approaches are dominant for LAR tracking as well as user detection and intent perception. In this case, the user must always stay within the camera’s field of view and carry multiple sensors for interaction. Thus, we adopted a solution that can simultaneously track the LAIDR and the user and enable the user to perform dual interactions with only a single wearable sensor anywhere inside the larger space rather than remaining near the LAIDR. For example, the LAIDR can follow an actor’s position and capture movie shots inside a large theater, as highlighted in Figure 1.

An essential requirement for HRI with a LAIDR is to absolutely localize both the vehicle (LAIDR) and dynamic target (user). For LAIDR flight control, the absolute heading angle measurement provides an additional requirement. Several indoor tracking technologies are available, with their benefits and disadvantages. Among them, motion capture systems such as Optitrack or Vicon are the best tracking systems for high pose (position and heading) accuracy and multi-target tracking [17,18,19]. However, these tracking systems are; very expensive as space area increases, sensitive to ambient light conditions, complex to install, and require proper calibration. SLAM and deep-learning-based localization are other popular approaches that have shown good results [20,21,22,23]. However, these systems are confined to a local reference frame and usually require sophisticated hardware, e.g., vision sensors such as a laser scanner, depth camera, and monocular camera, which require complex and computationally expensive algorithms. Moreover, because of their narrow field of view, short range, and the occlusion issue of the vision sensors, they cannot track multiple targets throughout a larger indoor space. An inertial navigation system (INS) fused with other sensors can track the position of an ego vehicle in a large indoor area [24,25]. However, this system lacks multi-target tracking and pose estimation, especially the heading, and suffers from indoor electromagnetic instability owing to the inclusion of a magnetometer.

To overcome the shortcomings of the abovementioned tracking systems, we introduced a UWB-based tracking system in this work, which is not only inexpensive but also easy to deploy in a large indoor space without mapping the surrounding surfaces [26]. In essence, the inclusion of a UWB sensor network fulfills the tracking needs of our system in an indoor environment. Firstly, it helps us to achieve a long enough range and lower coverage cost per operation area than a vision-based system. Secondly, it enables us to track the LAIDR position and the user position independent of indoor ambient light conditions, as the UWB sensors operate using radio frequency (RF) signals [27]. Thirdly, it allows us to perform HRI by detecting user intent either by their positional changes or by their on-air arm gestures anywhere inside the wider space. Finally, using multiple UWB sensors in the LAIDR permits us to compute the absolute heading angle instead of using a Pixhawk4 magnetometer. Thus, excluding a magnetometer renders the LAIDR state estimation less prone to varying indoor electromagnetic intensity.

Usually, when it comes to flying vehicles’ autonomous flight, Pixhawk4 is a popular option as the autopilot board. However, Pixhawk4 requires special integration for both the sensory feedback/data-sharing and the control system when the flying vehicle is custom-built such as our LAIDR platform. The sensory data-sharing is straightforward involving simple attachment of the GPS receiver at the respective serial port of the Pixhawk4 when the flying vehicle is in operation outdoors [12]. Indoors, which is characterized as a GPS-denied environment, data-sharing with the Pixhawk4 is highly dependent on the sensor type. Typically, indoor tracking for drones is achieved using a motion capture system [18,28], where the micro air vehicle communication protocol on the ROS framework (MAVROS) helps share the external sensory data with Pixhawk4. We responded with a similar approach, but the sensor measurement is acquired by the UWB sensor network. In addition, the control system of commercial off-the-shelf drones is based on the standard PX4 flight stack owing to the availability of their airframes inside this stack [29,30,31]. However, the same stack could not be adopted for controlling our custom-built LAIDR. Therefore, in contrast to previous works, we utilized open-support platforms, e.g., MATLAB/Simulink PX4 autopilot support from the UAV Toolbox [32], to build a real-time flight control algorithm.

In the recent past, we have performed few studies using the UWB sensor network for indoor applications [33,34,35]. In [33], we used the UWB to localize the ground robot and a user in a narrower indoor space, where a user does not perform a direct interaction with the robot but with the augmented content. In addition, in [34,35], the scope of the studies were limited only to track the LAIDR’s position using the UWB. In this work, we propose and demonstrate the UWB-based HRI between the LAIDR and a user in a wider indoor space, for the first time to the best of our knowledge. This study provides a facility for a user interaction with a flying vehicle before and during the flight without user-interactive distance constraints. The main contributions are summarized as follows:

- We developed the data processing scheme over an ROS framework to accommodate the custom-built LAIDR [34] platform’s integration needs for the user-centered application. In addition, we incorporated the UWB sensor network to overcome the influence of the indoor environment’s uncertainties on the LAIDR’s pose and user’s position tracking simultaneously;
- We proposed the dual interactions to exhibit the user-centered autonomous following of the LAIDR anywhere inside the operational space by relying only on the user’s 3D positional data acquired by their hand-held UWB sensor. The first interaction revealed a responsive update for the LAIDR’s path to autonomously follow the user footprint (2D position), while the second interaction detected the user intention by recognizing their on-air gesture (a spatial pattern) using a deep learning model.

The paper is organized into the following six sections. Section 2 describes the system architecture and methods. The experimental setup is explained in Section 3. The experimental results are listed in Section 4. The discussion appears in Section 5. A conclusion is provided in Section 6.

## 2. System Architecture and Methods

This section explains the vehicle platform and control system. Moreover, it portrays the indoor tracking system for the LAIDR pose and user position. Finally, it elaborates the data processing scheme required for the UWB-based LAIDR integration in a user-centered interactive application.

### 2.1. Vehicle Platform and Control System

#### 2.1.1. Specifications

**Figure 2:**
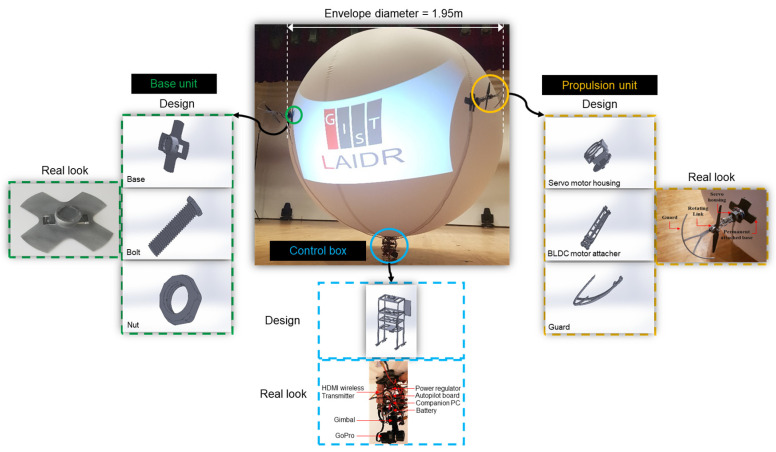
Detailed view of the LAIDR body and accompanying 3D-printed parts.

Our vehicle is a spherical body helium inflated robot named LAIDR, as shown in Figure 2, and its specifications are provided in Table 1. Helium gas was used to inflate the spherical hull to 1.95 m diameter, canceling the overall payload of 3.833 kg and producing extra lift equivalent to 0.139 kg payload [36]. The LAIDR was equipped with four propulsion units (PUs), which were divided into two pairs and distributed around the LAIDR’s great-circle at 0°, 90°, 180°, and 270°, and each pair held a different driving function. Each PU comprised a brushless direct current (BLDC) motor and a servo motor, and possessed a 0° to 180° tilt around the central axis.

#### 2.1.2. Motions

In the current design, the 3 DOF motions are controlled including the linear motions over the local X-axis and Z-axis combined with the ability to rotate around the local Z-axis. PU_1_ and PU_2_ are responsible for vertical motion by keeping the height setpoint, thus applying a vertical force on the balloon body to push it up or down. Whereas PU_3_ and PU_4_ are responsible for horizontal motion by controlling the heading angle to generate forward or backward motions, breaking, and allowing spinning around the local Z-axis in both directions. Let us consider $I_{HM}$ as the control input to move horizontally using PU_3_ and PU_4_, and $I_{VM}$ as the control input to perform vertical motion using PU_1_ and PU_2_. Equations (1)–(5) show the $I_{HM}$ and $I_{VM}$

(1) $$I_{HM}=\left[\begin{array}{c} F_{X}\\ \tau_{Z} \end{array}\right]=\left[\begin{array}{c} \left(F_{M3}{+F}_{M4}\right)\times D_{HM}\\ {B\times (F}_{M3}-F_{M4})\times R_{HM} \end{array}\right]\;,$$

(2) $$D_{HM}=\{\begin{array}{c} Forward;\;when\;\psi_{S3}=0{}^{\circ}\;and\;\psi_{S4}=0{}^{\circ}\\ Backward;\;when\;\psi_{S3}=180{}^{\circ}\;and\;\psi_{S4}=180{}^{\circ} \end{array}$$

(3) $$R_{HM}=\{\begin{array}{c} Left;\;when\;\psi_{S3}=0{}^{\circ}\;and\;\psi_{S4}=180{}^{\circ}\\ Right;\;when\;\psi_{S3}=180{}^{\circ}\;and\;\psi_{S4}=0{}^{\circ} \end{array}$$

(4) $$R_{HM}I_{VM}{=F}_{Z}=\left(F_{M1}{+F}_{M2}\right)\times D_{VM}\;,$$

(5) $$D_{VM}=\{\begin{array}{c} Upward;\;when\;\psi_{S1}=0{}^{\circ}\;and\;\psi_{S2}=0{}^{\circ}\\ Downward;\;when\;\psi_{S1}=180{}^{\circ}\;and\;\psi_{S2}=180{}^{\circ} \end{array}$$

where $F_{X}$, $F_{Y}$, and $F_{Z}$ are the force required for LAIDR’s movement over XYZ-axes and $\tau_{Z}$ represents the torque required to rotate LAIDR around the Z-axis. $F_{M1}$, $F_{M2}$, $F_{M3}$, and $F_{M4}$ are the propulsion forces generated by BLDC motors (*M*1, *M*2, *M*3, and *M*4), respectively. $\psi_{S1}$, $\psi_{S2}$, $\psi_{S3}$, and $\psi_{S4}$ refer to the rotating angle generated by servo motors (*S*1, *S*2, *S*3, and *S*4), respectively. The constant term B is the radius of LAIDR’s hull, i.e., *B* = diameter/2. ${\;D}_{\mathrm{HM}}$ defines the direction of horizontal motion and $R_{HM}$ expresses the rotation direction around the local Z-axis. $D_{VM}$ represents the direction of vertical motion.

#### 2.1.3. Control System

To design the autopilot’s control algorithm for the LAIDR, we used the PX4 Autopilot Support for UAV Toolbox provided by MathWorks [32] as the development platform. This toolbox supports building customized real-time flight control and vehicle management algorithms.

As mentioned earlier, in the current design of the LAIDR, we aimed to control the linear motions over the local X-axis and Z-axis and the rotational motion around the local Z-axis. Thus, models representing each of them required development to control these motions. Therefore, we built different motion models using the MathWorks System Identification toolbox in the form of S-domain transfer functions [12]. Although we represented the LAIDR’s motions in a linear form in order to tune conventional proportional integral differential (PID) controllers, this linear representation was sufficient to maintain stability while flying such a non-linear system.

**Figure 3:**
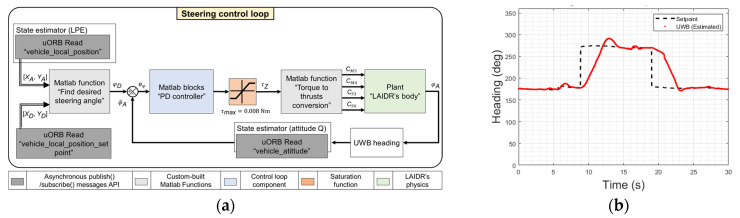
Steering controller: (**a**) block diagram; (**b**) performance.

The first model represents the change in LAIDR’s heading, where we passed the torque data $\tau_{Z}$, while spinning around the Z-axis, as an input and stored the change in the heading angle around the same axis $\dot{\varphi }$ as an output of this model. $\tau_{Z}$ was calculated using (1), and $\dot{\varphi }$ was stored using the Pixhawk4 autopilot’s on-board sensors. Following (1), we operated *M*3 and *M*4 at different speeds to generate various spinning torque values and recorded $\dot{\varphi }$. Both *M*3 and *M*4 remained in the home position “facing forward” to prevent generating horizontal or vertical motions while collecting the data. A marginally stable third-order transfer function was the output of the identification process. Accordingly, we tuned the PD controller parameters to stabilize the system performance for the desired angle input. Figure 3a shows the design of the steering control loop. The error $e_{\varphi }$ was generated from the difference between the desired $\varphi_{D}$ and the estimated ${\hat{\varphi }}_{A}$ UWB heading angles of the LAIDR’s body. The commanded torque $\tau_{C}$ was the output of the steering controller and the input for the “Torque to thrust conversion” MATLAB function. This function generated the thrust amounts for *M*3 and *M*4 as $C_{M3}$ and $C_{M4}$ signals, respectively, and the desired rotation for *S*3 and *S*4 as $C_{S3}$ and $C_{S4}$ signals, respectively. We checked the steering controller performance by conducting a practical experiment ordering the LAIDR to follow a set of desired heading angles at three stages, as shown in Figure 3b. The result reflected a steady-state error of under ±2.0°. Besides the heading angle, the forward speed was also controlled during the horizontal motion using a concept similar to the one introduced in [34].

**Figure 4:**
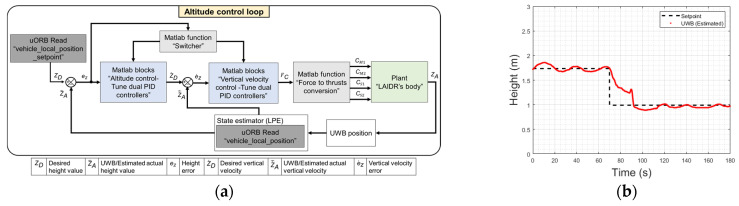
Altitude controller: (**a**) block diagram; (**b**) performance.

To control the LAIDR height, we developed an altitude-changing model following similar steps. To develop this model, we measured the vertical applied force in (4) and passed it as the model’s input, then recorded the vertical displacement using the UWB positioning sensor, which was considered as the model’s output. Only *M*1 and *M*2 were activated and faced upward or downward while the other two motors were disabled. This guaranteed a pure vertical displacement without encountering any horizontal or rotational movements. The two identified models for ascending and descending helped tune a pair of PID controllers to track the change in the desired altitude input. We designed the altitude control loop with a pair of cascaded controllers for robust tracking of the desired height under a controlled ascending or descending velocity [14], as shown in Figure 4a. According to the flying direction, different pairs of controllers are activated by the “switcher” function to adapt the different behaviors of the LAIDR when ascending or descending. Through the “Force to thrust conversion” MATLAB function, the commanded vertical force $F_{C}$ is decomposed to thrust for *M*1 and *M*2 as $C_{M1}$ and $C_{M2}$ signals, respectively, and the desired rotation for *S*1 and *S*2 as $C_{S1}$ and $C_{S2}$ signals, respectively. To check the altitude controller performance, we intended making a sudden change in the LAIDR’s desired height after maintaining a stable height. Figure 4b shows the practical performance for the LAIDR to descend from 0.17 to 0.99 m height setpoints. The altitude controller clearly regulated the descending operation to compensate for the inertial behavior of the LAIDR body. The result showed an average height tracking error of under ±0.15 m throughout the conducted experiment.

### 2.2. Indoor Tracking System for Vehicle Pose and User Position

#### 2.2.1. UWB Sensor Network and Data Acquisition

**Figure 5:**
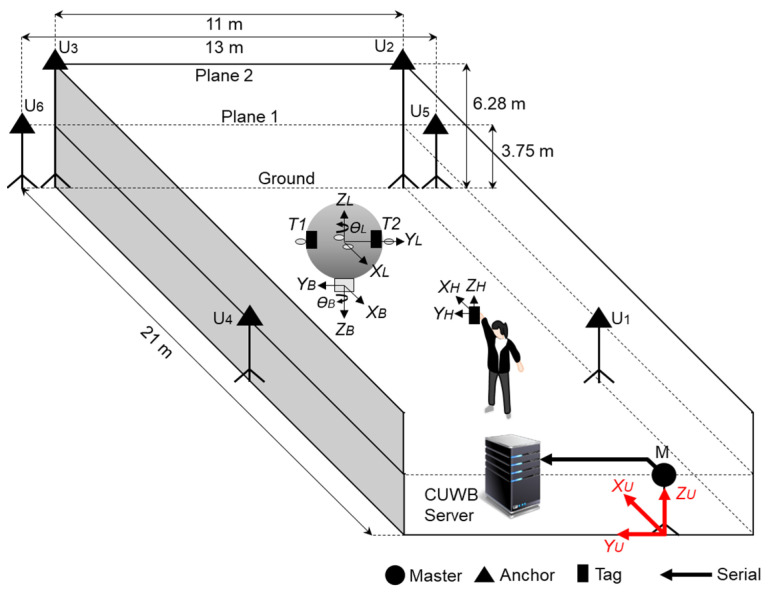
Deployment of the UWB sensor network to track the LAIDR’s pose and the user’s position.

The UWB sensor network, as constructed by the Ciholas [37], was used as the basis for the absolute positioning system to track the LAIDR and user. The Ciholas UWB sensor network is a combination of many modules with uniform hardware configuration mainly based on a Decawave’s DW1000 transceiver and an ARM’s 32-bit Cortex-M4 processor. Each module can be assigned to multiple operation modes such as master, anchor, and tag. The master and anchor are static modules while the tag is a movable module. In this work, the infrastructure of the seven modules was installed, i.e., six as anchors and one as master. These modules were attached to either the walls or tripods. For 3D positioning, the anchors were placed in two planes above ground, i.e., plane 1 and plane 2. Moreover, three tags were placed, i.e., two on LAIDR and one on the user. Figure 5 illustrates the deployment of the UWB sensor network, and the 3D coordinates of the infrastructure are provided in Table 2.

Data acquisition from the UWB sensor network was achieved by connecting the master to the desktop via serial port where the Ciholas ultra-wideband (CUWB) server software Archimedes version 2.0 [38] was installed. The CUWB server performed real-time location system (RTLS) calculations and broadcast the location data of the moving agents to external applications over UDP. Moreover, it combined the UWB network manager and graphical user interface (GUI) and enabled users to access the UWB infrastructure configuration as well as the visualization of the location data. The standard position accuracy of Ciholas RTLS is about ±0.10 m laterally and ±0.20 m vertically. In addition, a tag with 10 Hz sample rate consumes 150 mW at 5 V [37], therefore it can last up to 16.40 h with a smaller battery of 500 mAh capacity.

#### 2.2.2. Coordinate Frames

The characterization of the coordinate frames displayed in Figure 5 are as follows:

- ($X_{U},\;Y_{U},Z_{U}$) is the UWB frame or the world frame;
- ($X_{L},\;Y_{L},Z_{L},\theta_{L}$) and ($X_{B},\;Y_{B},Z_{B},\theta_{B}$) are the two representations of the LAIDR platform’s pose in the UWB frame and local PX4 frame, respectively. Section 2.2.3 details the LAIDR’s UWB-based pose calculation and Section 2.2.4 specifies the UWB-based pose frame conversion from the UWB frame to the local PX4 frame;
- ($X_{H},\;Y_{H},Z_{H}$) is the user’s position in UWB frame.

#### 2.2.3. UWB-Based LAIDR Pose Calculation

**Figure 6:**
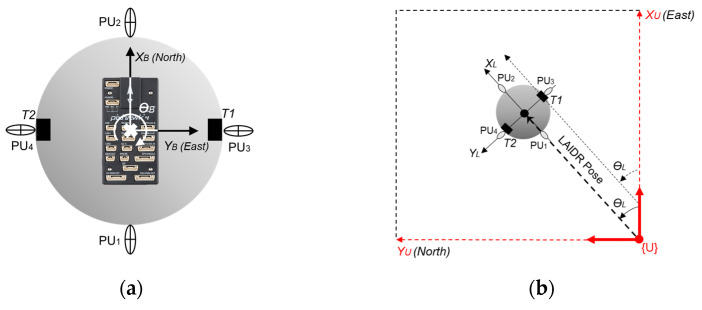
The LAIDR’s pose computation using the UWB sensor network. (**a**) placement of autopilot board, UWB tags, and propulsion units on the LAIDR in the local PX4 frame; (**b**) the LAIDR’s heading in the UWB frame.

The pose measurement of the LAIDR is a basic requirement prior to perform navigation application. The absolute position as well as heading of the LAIDR are computed using only the UWB sensor network. Where the two tags, i.e., tag 1 (*T*1) and tag 2 (*T*2), are placed at the edges of the LAIDR’s hull near the PU_3_ and PU_4_, respectively. The tracked position coordinates of these tags are known and provided by the CUWB server. As these tags are not in the center of the LAIDR, therefore, the displacement between them has to be accounted for. The average value of tags’ position coordinates is considered as the LAIDR position while the relative difference corresponds to the LAIDR heading. Figure 6 describes the tags’ placement at the LAIDR and the LAIDR pose computation in the UWB frame. The UWB-based LAIDR pose can be deduced as

(6) $$P_{L}=\left[\begin{array}{c} X_{L}\\ Y_{L}\\ \begin{array}{c} Z_{L}\\ \theta_{L} \end{array} \end{array}\right]\;,$$

(7) $$X_{L}=\frac{\left(x_{T1}+x_{T2}\right)}{2}\;,\;\;Y_{L}=\frac{\left(y_{T1}+y_{T2}\right)}{2}\;,\;\;Z_{L}=\frac{\left(z_{T1}+z_{T2}\right)}{2}\;,$$

(8) $$\theta_{rad}=atan2\left(y_{T1}-y_{T2},\;x_{T1}-x_{T2}\right)\in \left(-\pi ,\;\pi \right)\;,$$

(9) $$\theta_{L}=\{\begin{array}{c} \theta_{rad};\;\;\;\;\;\;when\;\theta_{rad}\geq 0\\ \theta_{rad}+2\pi ;\;when\;\theta_{rad}<0 \end{array}$$

where $P_{L}$ is the LAIDR pose, ($X_{L},\;Y_{L},Z_{L}$) are the LAIDR position coordinates, and $\theta_{L}$ is the heading (bearing angle) of the LAIDR between the *T*1 and *T*2 in radian. While ($x_{T1},y_{T1},\;z_{T1}$) and ($x_{T2},y_{T2},z_{T2}$) are the position coordinates of the *T*1 and *T*2, respectively. All values are considered with respect to the UWB frame.

#### 2.2.4. Pose Fusion with PX4 Flight Stack

**Figure 7:**
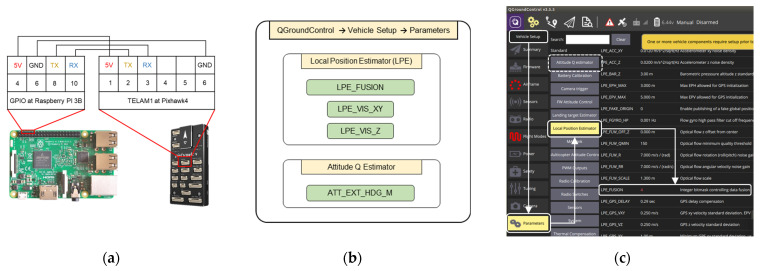
UWB-based LAIDR pose fusion with PX4 Flight Stack: (**a**) physical connection between a companion computer and an autopilot board; (**b**) parameters’ path; (**c**) description of the “LPE_FUSION” value at the QGC.

Usually, a PX4 flight stack relies on the GPS sensor in the outdoor environment and realizes the vehicle state (position and velocity estimates) via default settings. As in this work, the UWB sensor substitutes the GPS sensor for the LAIDR’s localization in the indoor environment, therefore a dedicated ROS package (Section 2.3) and a physical connection (Figure 7a) are established to share the LAIDR UWB pose with the PX4 flight stack. In detail, we followed the external pose input documentation provided by the PX4 team [39] and set the two parameters named as “LPE_FUSION” and “ATT_EXT_HDG_M” of the PX4 flight stack to “4” and “Vision”, respectively. These parameters activate the inner state estimators such as the local position estimator (LPE) and attitude Q (quaternion) estimator. The LPE and Q (LPEQ) are based on an extended Kalman filter to estimate the LAIDR’s position and velocity, and a complementary filter to estimate the LAIDR’s orientation [40], which means the LPEQ apply the sensor fusion between the external, i.e., UWB, and internal, i.e., 6 DOF IMU, sensors. Hence, the estimated results of the LPEQ are used to update the pose of the autopilot board as the LAIDR’s current state in the local PX4 frame. Moreover, we tuned the two noise parameters named as “LPE_VIS_*XY*” and “LPE_VIS_Z” with the values of “0.01” and “0.04”, respectively, according to the UWB standard position accuracy (Section 2.2.1). Figure 7b,c illustrates the required parameters’ path and setting at the QGroundConrol (QGC) [41].

After parameters’ setting, the companion computer publishes the raw UWB-based LAIDR’s pose (3D position and heading) as an ROS message with the definition of “geometry_msgs/PoseStamped.msg” [42] and the autopilot board’s LPEQ subscribe this message. Equations (10)–(12) show the uploaded raw UWB measurements to an ROS message

(10) $$PoseStamped=\left[\begin{array}{c} pose.position\\ pose.orientation \end{array}\right]\;,$$

(11) $$pose.position=\left[\begin{array}{c} X_{L}\\ Y_{L}\\ Z_{L} \end{array}\right]\;$$

(12) $$pose.orientation=\left[\begin{array}{c} Q_{x}\\ Q_{y}\\ \begin{array}{c} Q_{z}\\ Q_{w} \end{array} \end{array}\right]=\left[\begin{array}{c} 0\\ 0\\ \begin{array}{c} \sin\left(\frac{1}{2}\theta_{L}\right)\\ \cos\left(\frac{1}{2}\theta_{L}\right) \end{array} \end{array}\right]$$

where PoseStamped represents the UWB-based LAIDR’s pose, pose.position is the UWB-based LAIDR’s 3D position and pose.orientation is the UWB-based LAIDR’s heading defined as a quaternion ($Q_{x},\;Q_{y},Q_{z},Q_{w}$).

It is to be noted that the UWB-based LAIDR’s pose in the world frame follows the east north up (ENU) convention which is a standard ROS convention and the LAIDR’s state in the local PX4 frame follows the north east down (NED) convention. The MAVROS performs the frame transformation from ENU to NED automatically, so there is no need to transform the frame manually.

### 2.3. Data Processing Scheme

**Figure 8:**
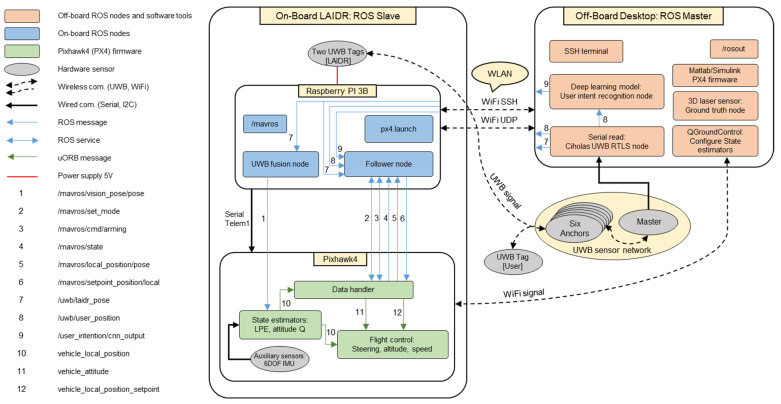
Data processing scheme using the ROS framework.

This section highlights the complete data processing pipeline required by the LAIDR’s custom-built hardware as shown in Figure 8. The detail of the off-board and on-board data processing subsections is illustrated in the following subsections.

#### 2.3.1. Off-Board

A desktop computer with Ubuntu 16.04-LTS and an ROS Kinetic environment was selected as an off-board data processing unit. All the necessary software tools such as MATLAB 2019a with the supported toolboxes (Simulink, PX4 autopilot support from UAV Toolbox) [32], CUWB server [38], QGC [41], 3D laser sensor [43] ROS package, and Anaconda [44], etc., were installed on the desktop computer. Firstly, the MATLAB/Simulink was used to develop the feedback controller algorithm, debug, and generate the binary code as firmware for downloading into Pixhawk4. Secondly, the CUWB server was used to collect time-of-flight data, calculate tags positions, and export the UWB positions over the UDP for external applications. Then, the Ciholas RTLS node imports the UWB positions over UDP and published the LAIDR’s UWB pose and the user’s UWB position as separate ROS topics, i.e., /uwb/laidr_pose and /uwb/user_position. Thirdly, the QGC was utilized to calibrate all the embedded auxiliary sensors, to configure the sensor fusion at Pixhawk4, and to monitor the LAIDR in-flight state information. Fourthly, the 3D laser sensor ROS node was utilized to extract and store the point cloud data for the 3D ground truth measurement. Finally, Anaconda was employed to train and test the deep learning classifier for user intent recognition and publish it as an ROS topic, i.e., /user_intention/cnn_output.

#### 2.3.2. On-Board

This mainly consisted of two sub-processing units, the Raspberry PI 3B as the companion computer and Pixhawk4 as the autopilot board. The companion computer had a Raspbian image which is running on top of an Ubuntu 16.04 LXDE and integrated with the ROS Kinetic, while the autopilot board was incorporated with the PX4 flight stack [30]. The bridge between the companion computer and autopilot board had a standard ROS package called MAVROS [45], which supported the MAVLINK protocol and established a seamless bilateral communication between the ROS-enabled computer and the PX4 flight stack-enabled autopilot board. Moreover, the PX4 flight stack used the MAVLINK over a WiFi signal to communicate with the off-board QGC. Specifically, the main job of the companion computer was to process the tasks that could not be executed at the autopilot board; more precisely, it received the external sensory information, i.e., the UWB pose of the LAIDR and the UWB position of user, and generated the waypoints as per user interaction. The autopilot board performed the tasks such as state estimation and feedback control to stabilize the LAIDR’s flight.

To successfully implement the interaction with the UWB-based LAIDR, an ROS package containing two ROS nodes was designed. The first ROS node, i.e., UWB fusion node, described the LAIDR’s UWB pose, and published an ROS message of ROS topic, i.e., /mavros/vision_pose/pose, to fuse the UWB pose at the autopilot board [39]. The second ROS node, i.e., follower node, presented the user’s UWB position and feedback LAIDR state for interactive decision making, and, accordingly, published the waypoint message, i.e., /mavros/setpoint_position/local, for the feedback control system at the autopilot board. In Figure 8, the ROS topics 1, 5, 6, 7, and 8 bear a message definition of “geometry_msgs/PoseStamped.msg” [42].

## 3. Experimental Setup

**Figure 9:**
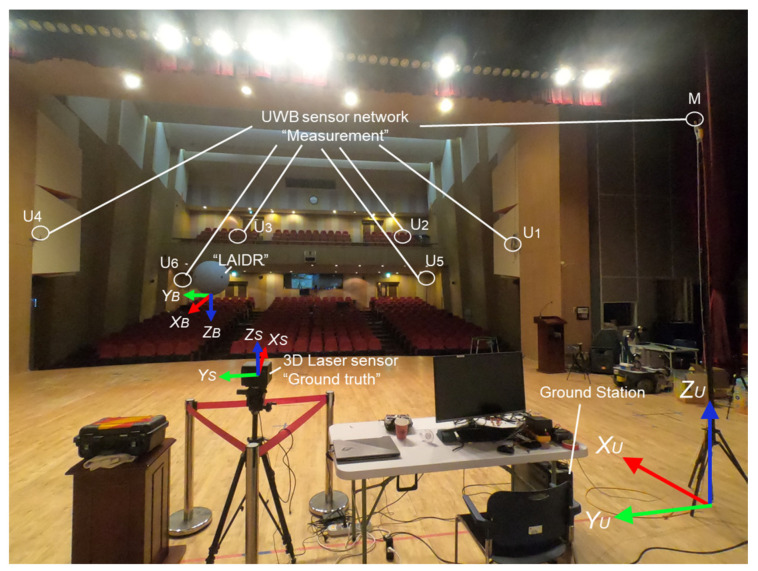
Photo of the experimental environment showing the UWB sensor network in the ENU frame, the LAIDR platform in the NED frame, the 3D laser sensor in the ENU frame, and the ground station.

To evaluate the proposed system performance, the experimental equipment was setup in the Art Hall facility at the Gwangju Institute of Science and Technology. This facility is a professional indoor arena for hosting cultural and social exhibitions. An elaboration of the experimental environment is shown in Figure 9.

A total of two experiments were carried out. The first experiment reflected the evaluation of the UWB-based LAIDR pose accuracy and the autonomous following based on the pre-assigned waypoints. The second experiment described the user-centered autonomous following of the LAIDR platform where the variable waypoints were designated based on the user footprint and user intention (up to the classification stage). For these experiments, the average LAIDR speed was 0.5 m/s and the sample rates of the data, such as the LAIDR state, user position, and ground truth position, were adjusted to 10 Hz. For the later analysis, all the data were recorded as a ROSBAG file on the desktop.

### 3.1. UWB-Based LAIDR Pose Tracking and Fixed Waypoints Following

#### 3.1.1. Scenario

**Figure 10:**
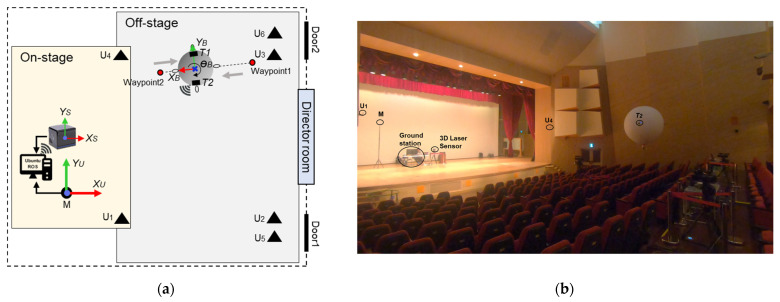
Pre-assigned waypoints following setup: (**a**) scenario layout; (**b**) the LAIDR during the experiment at 1 s instant.

This experiment evaluated the UWB-based LAIDR’s pose tracking performance while following the pre-assigned waypoints. More precisely, a constrained straight-line motion alternated between the two fixed waypoints, i.e., (X = 17.4 m, Y = 7.5 m) and (X = 10.6 m, Y = 6.7 m) were established using the UWB pose measurement as feedback. Where the desired height was kept constant at 2.5 m and the threshold of a 0.6 m circle radius was used for the waypoints registration. Moreover, a 3D laser sensor was considered to validate the UWB-based LAIDR’s 3D position accuracy, and the trace motion was used to assess the UWB-based LAIDR’s heading angle as the 3D laser sensor cannot measure the heading angle. Figure 10 shows the scenario layout and the LAIDR flying during the experiment.

#### 3.1.2. Ground Truth Position Computation and Transformation

**Figure 11:**
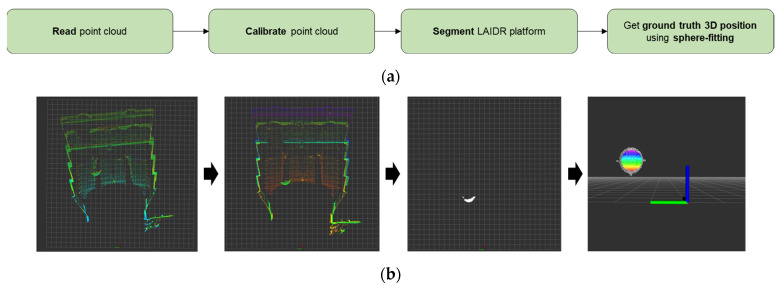
Ground truth position computation using 3D laser sensor’s point cloud: (**a**) implementation steps; (**b**) visual output of each step.

To confirm the tracking accuracy of the UWB sensor network during the LAIDR flight, a 3D laser sensor [43] with 170 m range, ±30 mm accuracy, and 110° horizontal and 20° vertical field of view was deployed. Before extracting the ground truth 3D position of the LAIDR, the point cloud of the 3D laser sensor was calibrated against the roll, pitch, and yaw offsets. Subsequently, only the LAIDR point cloud was segmented considering the operational cubical region. Next, the sphere-fitting technique [46] was applied over the segmented LAIDR point cloud to compute the ground truth 3D position. The implementation steps and respective visual output are shown in Figure 11.

For validation purposes, we deployed a 3D laser sensor as a ground truth positioning system. As described in Section 2.2.2, the LAIDR position is computed with respect to UWB frame {U} which is equivalent to the world frame. Thus, the coordinate frame of 3D laser sensor {S} should be transformed to {U}, which is given as

**Figure 12:**
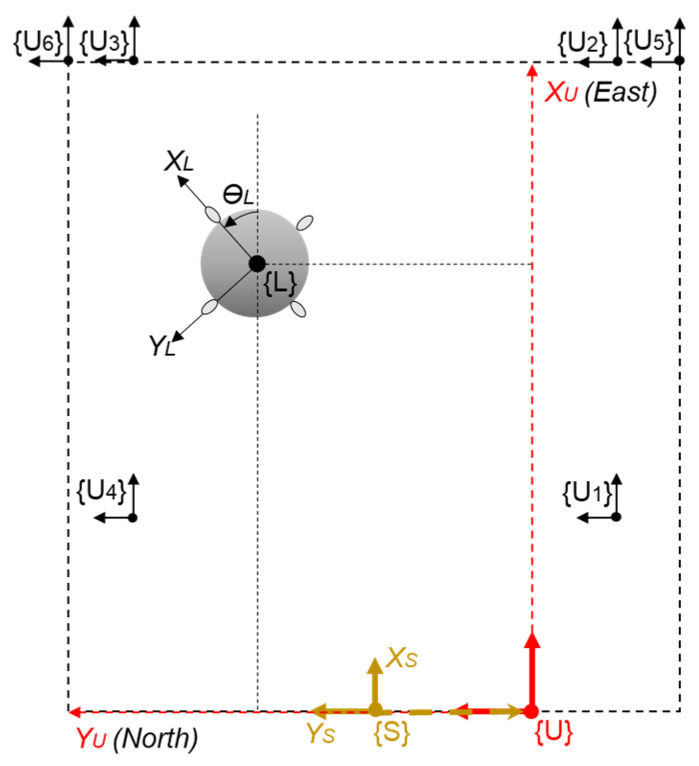
Coordinates transformation of the ground truth position.

(13) [PSLW1]=TSW[PSLS1], TSW=[1000010000100dYS01], dYS=YL−YS,

where PSLW and PSLS are the LAIDR ground truth position coordinates in {U} and {S}, respectively, $d_{YS}$ is the translation in Y-axis and TSW is the transformation matrix for the LAIDR’s ground truth position transformation from {S} to {U}. Figure 12 visually highlights the coordinate frames transformation of the 3D laser senor.

### 3.2. User-Centered Autonomous Following

#### 3.2.1. Based on User Footprint

This experiment demonstrated the applicability and repeatability of our proposed system when fitted for an indoor interactive application. In addition, it highlighted the long-range and multi-agent tracking abilities of the UWB sensor network to incorporate the user interaction with the LAIDR. The UWB position of the user specified their footprint as the target waypoint for the LAIDR’s autonomous following. This was a more precise way of forming a line trajectory as the user maintained their position and switching to another line as the user changed their position. The starting waypoint was the user’s real-time position and the end waypoint had the X-axis value of the same user position and the Y-axis value of the operation space area. Algorithm 1 expresses the alternation of real-time dynamic waypoints reacting to the change in the user position. Where the threshold of 4.0 m circle radius based on human visibility in the camera was considered for starting waypoints registration, while the values for the desired height and the threshold of the end waypoints registration were kept the same as experiment 1.
**Algorithm 1** Follower node execution flow for user-centered waypoints following**Input:** ROS topics “/mavros/local_position/pose” and “/uwb/user_position”, and operation space area **Output:** ROS topic “/mavros/setpoint_position/local” **Procedure:** Start with current user position as target waypoint **Subscribe** ROS topic “/uwb/user_position” **Update** “current user position” from ROS topic “/uwb/user_position” *i* = 2 waypoint (*i*) = (current user position X-axis, current user position Y-axis) threshold = 4.0 m while *(*true*)*      **Subscribe** ROS topic “/mavros/local_position/pose”      **Update** “current LAIDR position” from ROS topic “/mavros/local_position/pose”      **Subscribe** ROS topic “/uwb/user_position”      **Update** “current User position” from ROS topic “/uwb/user_position”      if (distance (current LAIDR position, waypoint (*i*)) ≤ threshold)      Switch the target waypoint      if (*i* = 2)         *i* = 1         waypoint (*i*) = (current user position X-axis, operation space Y-axis upper limit)         threshold = 0.6 m      else         *i* = 2         waypoint (*i*) = (current user position X-axis, current user position Y-axis)         threshold = 4.0 m      **Write** waypoint (*i*) to ROS topic “/mavros/setpoint_position/local”      **Publish** ROS topic “/mavros/setpoint_position/local”

**Figure 13:**
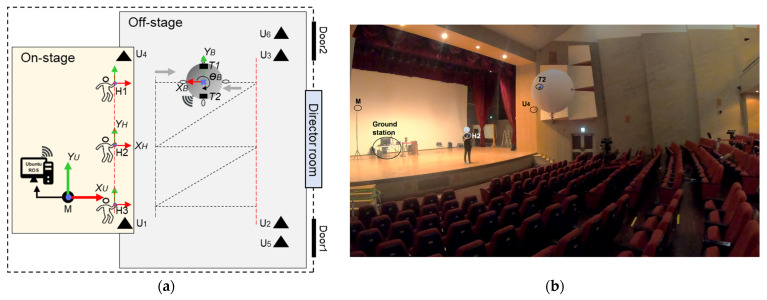
User-centered waypoints following setup: (**a**) scenario layout; (**b**) the LAIDR during the experiment and the user at the H2 location.

To reveal the applicability and repeatability of the proposed system during the user following, a total of three user positions (i.e., H1, H2, and H3) were considered. Figure 13 shows the scenario of user footprint-based waypoints following and the LAIDR flying alongside the user at the H2 position during the experiment. A demonstration video of this experiment is provided in [47]. In summary, the LAIDR supposed to autonomously follow the user footprint and form a zig-zag trajectory as the user held or varied his location, respectively, as shown in Figure 13a.

#### 3.2.2. Based on User Intention

**Figure 14:**
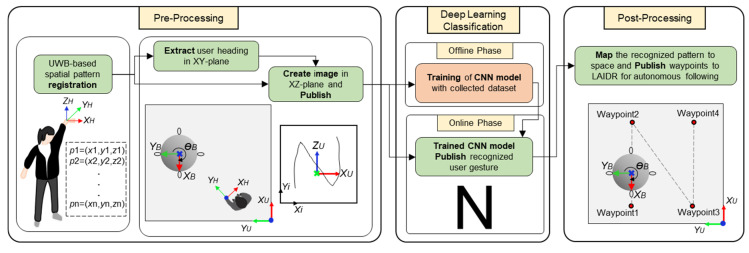
Concept illustration of user intention recognition based on his UWB-based on-air drawn spatial pattern.

In our proposed system, the user was equipped with a hand-held UWB tag so they had the freedom to draw on-air gestures in terms of a spatial pattern. This freedom enabled us to translate the user intention into a meaningful interaction with the LAIDR in a larger indoor space (e.g., the LAIDR can autonomously followed the same pattern). In this experiment, we elaborated upon this idea by classifying the letters and shapes. Figure 14 describes the concept and complete process, which was divided into three stages of pre-processing, deep learning classification, and post-processing.

**Figure 15:**
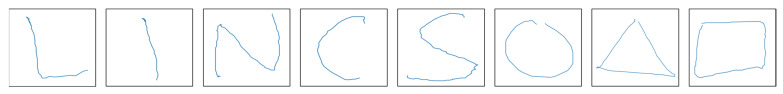
Sample dataset images created by Algorithm 2.

In the first stage, the data acquisition was explained in terms of the user’s on-air gesture registration and an input image creation for the deep learning classifier. The registration of the user’s gesture occurred during the period when the user pressed and released the button on the UWB tag [33]. The registered UWB-based on-air gesture consisted of the spatial 3D points. The key challenge was how to convert these points to an input image for the deep learning classifier. This challenge was tackled by apportioning them into two planes (i.e., XY and XZ). The 2D points lying over the XZ-plane were actually the projection of the desired input image but prone to the rotation offset across the Z-axis because of the user’s heading. Therefore, the 2D points in the XY-plane helped to measure this rotation offset. Algorithm 2 illustrates the detailed pre-processing stage including data acquisition and input image creation. As we wanted to translate the user intention into the LAIDR motion path, we therefore ended up selecting the eight on-air gestures under two types of shapes such as rounded (C, S, circle) and cornered (L, I, N, square, triangle). Figure 15 displays the sample input images of the user’s on-air gesture as the output of the pre-processing stage.
**Algorithm 2** Pre-processing execution flow**Input:** ROS topic “/uwb/user_position” **Output:** ROS topics “/user_intention/cnn_input_image” **Procedure:**      Initialize arrays “gesture[]” and “fgesture[]”,      while (true)           **1. UWB pattern registration:**           **Subscribe** ROS topic “/uwb/user_position”           Update “3DgestureXYZ” from ROS topic “/uwb/user_position”           if (button press == 1)              Append “gesture[]” by writing “3DgestureXYZ”           Apply an moving-average filter to smooth the stored array “gesture[]”           Update the “fgesture[]” after filter           **2. Rotation offset:**           Pick first and last point in XY-plane from “fgesture[]”           Get “rotation angle” by using inverse tangent on two points           **3. Input image:**           Calibrate the XZ-axes points from “fgesture[]” against “rotation angle” offset around Z-axis           Normalize the calibrated points as “input image” between min. and max. limits of XZ-axes           Write normalized image to ROS topic “/user_intention/cnn_input_image”           **Publish** ROS topic “/user_intention/cnn_input_image”

In the second stage, the deep learning-based classifier named convolutional neural network (CNN) is developed. The dataset (for training purpose) was made using single user’s on-air gesture simply by storing it with the label of each class. A total of 120 on-air gestures for each class were recorded. Table 3 describes the structure and parameters of the designed CNN model. The CNN model input was a feature group composed of an image in the XZ-plane. The spatial feature extraction layers contained three convolution layers, each with a 3 × 3 filter, 1 × 1 strides, and 32 depth, respectively. Every convolution layer was followed by a rectified linear unit (ReLU) activation function. In addition, three max pooling layers were used, each with a 2 × 2 filter, respectively. Then, the high-level characteristics were abstracted by a fully connected layer with batch normalization (BN) and a softmax function to obtain a probability for the final predictions.

In the third stage, an HRI application could be integrated as per user assistance in a wide indoor space. For example, the LAIDR can autonomously move in the same pattern, where the follower node is supposed to generate the waypoints according to the user intention output by the second stage. Before publishing to the LAIDR, these waypoints should be shifted and scaled across the center position of the operation space.

## 4. Experimental Results

### 4.1. UWB-Based LAIDR Pose Tracking and Fixed Waypoints Following

**Figure 16:**
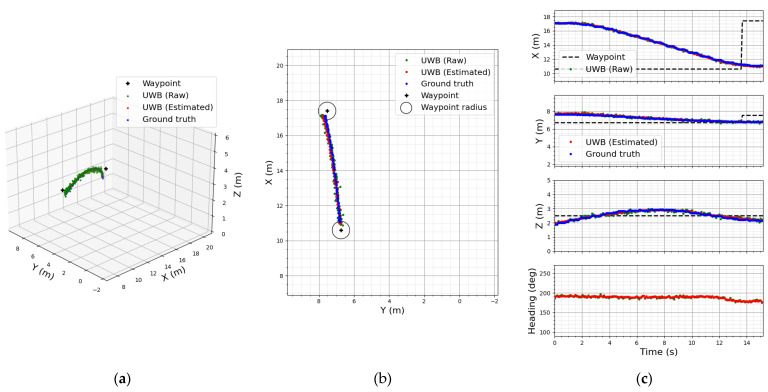
Fixed waypoints following results: (**a**) 3D plot; (**b**) 2D plot; (**c**) 1D plot with respect to time.

The resulting plots of this experiment are given in Figure 16. Figure 16a,b shows the LAIDR trajectory in terms of the raw UWB position (green dot), estimated UWB position (red dot), and ground truth (blue dot) position values in 3D and 2D, respectively. The LAIDR took almost 15 s to complete the trace shown in Figure 10a. The UWB-based LAIDR position errors compared to the 3D laser sensor are clearly visible in the 1D plots of X-axis, Y-axis, and Z-axis shown in the first three subplots of Figure 16c.

The bottom subplot of Figure 16c describes the raw and estimated UWB heading values of the LAIDR. Moreover, it can be observed from a heading angle between 12 s and 13.5 s, as the LAIDR approached the current target waypoint, its speed gradually decreased until braking and adjusted the heading angle before moving toward the next target waypoint. We used the 3D laser sensor for the quantitative evaluation of the UWB-based LAIDR 3D position accuracy which is highlighted as the root mean square error (RMSE) values in Table 4.

It is visually obvious from Figure 16b that the UWB-based LAIDR estimated trajectory was close to the desired trace between the two pre-assigned waypoints. In brief, this experiment laid the basis for integrating the UWB-based LAIDR for user-centered interactive application.

### 4.2. User-Centered Autonomous Following

#### 4.2.1. Based on User Footprint

**Figure 17:**
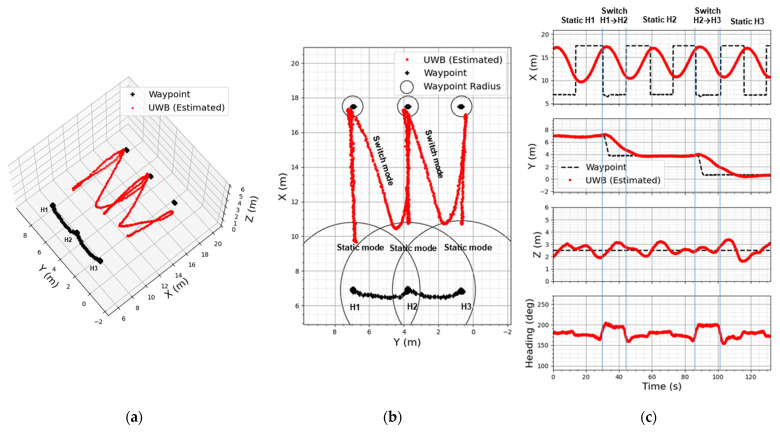
User-centered waypoints following results: (**a**) 3D plot; (**b**) 2D plot; (**c**) 1D plot with respect to time.

The user footprint-based waypoints following results are portrayed in Figure 17 as 3D, 2D, and 1D plots. The 2D plot in Figure 17b shows how stably the LAIDR reacted to three user positions by forming three straight and two diagonal lines, and Figure 17c illustrates the in-depth plots for each XYZ-axes and heading value.

To better understand the results according to static and switch modes of user interaction, the Figure 17c plots are divided into five time segments:

1. From start to 30 s when the user stays at the H1 position (static mode);
2. Above 30 s to 45 s when the user switches his position to H2 (switch mode);
3. Above 45 s to 87 s when the user holds the H2 position (static mode);
4. Above 87 s to 102 s when the user switches his position to H3 (switch mode);
5. Above 102 s to end when the user maintains the H3 position (static mode).

The alternation among the user footprint based waypoints and flight repeatability for segments 1−5 are clearly shown along the X-axis in the first subplot, while the smooth user following for segments 1,3,5 and the stable transition for segments 2,4 are shown along the Y-axis in the second subplot. This smooth performance in the transition between trajectories is due to damping of the rotational inertia using the whole operation range of both PU_3_ and PU_4_.

This experiment can be considered as a proof of applying the proposed UWB-based LAIDR to capture the user-specific automated video shoots (Figure 1).

#### 4.2.2. Based on User Intention

**Figure 18:**
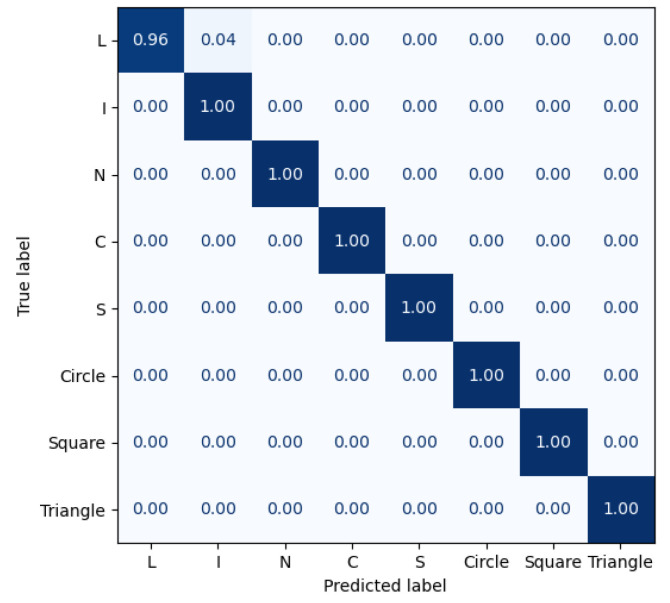
Confusion matrix of the CNN model trained on 8 classes.

The performance of the trained CNN model was validated using 20% of the dataset as a test sample. The results of the CNN model are shown as a classification report and a confusion matrix in Table 5 and Figure 18, respectively, where the presented CNN model accomplished 99.4% classification accuracy.

## 5. Discussion

Most of the available LAR platforms for indoor user-centered interactive applications retain a higher sensitivity to the indoor environment uncertainties (i.e., illumination intensity and electromagnetic field variations) and even lack the ability to detect and track distant user positions for interaction, as their tracking systems depend upon the camera and magnetometer sensors. This work aimed to express the aptness of our proposed system for the indoor user-centered interactive application. Thus, we conducted various experiments to reveal the vehicle’s 3D positioning accuracy, autonomous flight during fixed and user footprint-based waypoints following tasks, and the user’s intention perception by recognizing on-air drawn spatial pattern. Compared to [23], we accomplished a similar 3D positioning accuracy but over a wider area. At a glance, our results evaluated the overall waypoints following of the UWB-based LAIDR qualitatively. This time we validated the concept of the user intent perception by elaborating the offline phase (i.e., dataset creation in the pre-processing stage and training of the CNN model in the pattern recognition stage) although our dataset was based on only one user. However, it has provided sufficient evidence to extend our proposed idea in future with a more detailed analysis considering many users’ datasets and to complete the online phase by incorporating the post-processing stage. In contrast with [13,14,15], our proposed dual interactions could serve multiple HRI purposes at flexible interactive distances using the same indoor tracking system for the vehicle as well as the user and using a single wearable sensor.

**Figure 19:**
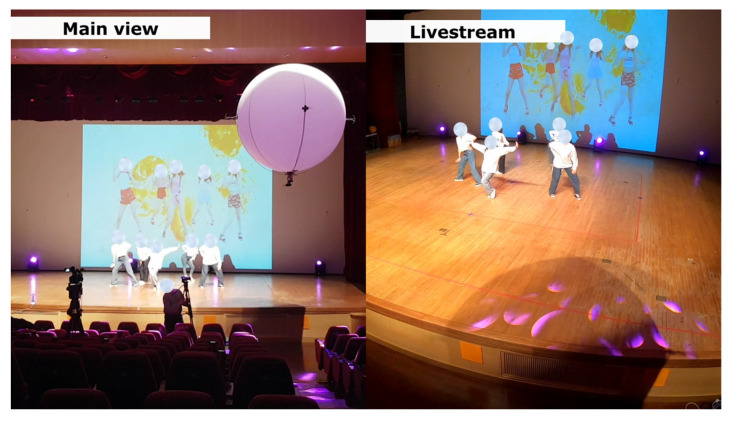
Demonstration of the UWB-based LAIDR as an indoor movie-making agent during a live event. (Left side: theater’s main camera view; right side: LAIDR’s GoPro shot).

We have successfully demonstrated a real-time use case for capturing movie shots of dancers inside a large theater (Figure 19 and video [48]) where the LAIDR platform autonomously followed the pre-assigned waypoints under the influence of the uncertainties of the indoor environment. In future, we intend to integrate our achievements in this work with the real-time application (Figure 1) by highlighting the ability of UWB-based LAIDR to autonomously follow the variable waypoints according to the proposed dual interactions.

Some points should be enhanced in future work for the sake of generalization. Firstly, the current control system is considered as linear which will be replaced with a non-linear control system. Secondly, the path planner will also be incorporated to exhibit the complex trajectory navigation using our proposed system. Thirdly, during experiments, we faced random delays in local WiFi communication, which disturbs the external UWB data sharing from the ground station to the LAIDR, affecting the LAIDR navigation performance. This can be addressed in future by processing the UWB position calculation on-board. Finally, although the UWB infrastructure deployment does not require mapping of surrounding surfaces; however, it would be a tedious job to manually measure the anchors coordinates especially when the indoor space bears an irregular geometry. In future, to fully benefit from the UWB sensor network ability, we wish to automatically compute the coordinates of the UWB infrastructure at the beginning.

## 6. Conclusions

In this paper, we proposed a UWB-based LAIDR as an autonomous system for user-centered interactive applications. Owing to concerns regarding indoor environment uncertainties, we integrated the UWB sensor network to simultaneously track the LAIDR pose and user position anywhere in a larger indoor space. We presented a complete data processing scheme for the in-house-developed LAIDR platform. We demonstrated a real-time use case for capturing movie shots of dancers inside a large theater where the UWB-based LAIDR autonomously followed the pre-assigned waypoints under a high-illumination light situation. To validate our proposed system, we conducted experiments where the waypoints following performance was qualitatively judged against the desired traces. The 3D position accuracy of the UWB-based LAIDR was verified using the 3D laser sensor as a ground truth system. Furthermore, we proved the applicability of the proposed dual interactions by first demonstrating the user-centered waypoints following and then using a CNN model to recognize the user intention. We are convinced that our findings will open up possibilities for the involvement of UWB-based robots, such as the LARs, in more autonomous and user-oriented indoor applications.

## Figures and Tables

**Table 1:** LAIDR component detail, function, and payload.

#	Item	Component Detail	Function and Quantity	Payload
1	Hull	PVC with 1.12 mm thickness	Spherical shape of 1.95 m diameter ×1	2.167 kg
2	Propulsion units	1. Hobbywing Ff-12AE	Motor driver ×4	0.460 kg
		2. T-Motor MT1306	BLDC motor ×4	
		3. HiTEC HS-5065MG+	Servo motor ×4	
3	Control box	1. Holybro Pixhawk4	Autopilot board ×1	0.612 kg
		2. Raspberry PI 3B	Companion PC ×1	
		3. NodeMCU ESP8266	LED music sync. over WiFi ×1	
		4. Arduino Pro-mini	Servo motors controller ×1	
		5. Battery 7.4 V, 2200 mAh	Power source ×1	
		6. Matec 5 V, 3 A	Power regulator ×1	
4	Positioning sensor	Ciholas DWUSB	UWB tag ×2	0.030 kg
5	Video streaming system	1. GoPro HERO7 BLACK	Camera ×1	0.388 kg
		2. Nyrius Aries Pro	HDMI wireless video transmitter ×1	
		3. FeiyuTech G6/WG2X	Gimbal ×1	
6	Cables/wiring	0.88 mm wires, MOLEX con.	Transfer power and control signals	0.176 kg

**Table 2:** 3D coordinates of the UWB sensor network’s infrastructure.

Value	M	U_1_	U_2_	U_3_	U_4_	U_5_	U_6_
X [m]	0.0	6.9	20.9	20.9	6.9	20.9	20.9
Y [m]	0.0	−2.2	−2.2	8.8	8.8	−3.7	10.3
Z [m]	3.75	3.75	6.28	6.28	3.75	3.75	3.75

**Table 3:** Structure and parameters of the CNN model.

Structure	Input	Filter	Depth	Stride	Output
Convolution + ReLU	288 × 432 ×3	3 × 3	32	1 × 1	286 × 430 × 32
Max Pooling	286 × 430 × 32	2 × 2	-	-	143 × 215 × 32
Convolution + ReLU	143 × 215 × 32	3 × 3	32	1 × 1	141 × 213 × 32
Max Pooling	141 × 213 × 32	2 × 2	-	-	70 × 106 × 32
Convolution + ReLU	70 × 106 × 32	3 × 3	32	1 × 1	68 × 104 × 32
Max Pooling	68 × 104 × 32	2 × 2	-	-	34 × 52 × 32
Fully Connected + BN	34 × 52 × 32	-	-	-	256
Softmax	256	-	-	-	10

**Table 4:** RMSE of the UWB-based LAIDR 3D position.

Value	RMSE
X [m]	0.08
Y [m]	0.06
Z [m]	0.05
2D [m]	0.10
3D [m]	0.11

**Table 5:** Classification report of the CNN model trained on 8 classes.

Class	Precision (%)	Recall (%)	F1-Score (%)	Support
L	100	96	98	23
I	96	100	98	23
N	100	100	100	24
C	100	100	100	23
S	100	100	100	23
Circle	100	100	100	24
Square	100	100	100	24
Triangle	100	100	100	24
Mean	99.5	99.5	99.5	Σ = 188

## Data Availability

The data presented in this study are available from the corresponding author upon reasonable request.
